# The application prospect of metal/metal oxide nanoparticles in the treatment of osteoarthritis

**DOI:** 10.1007/s00210-021-02131-0

**Published:** 2021-08-20

**Authors:** Junchao Luo, Yin Zhang, Senbo Zhu, Yu Tong, Lichen Ji, Wei Zhang, Qiong Zhang, Qing Bi

**Affiliations:** 1 Department of Orthopedics, Zhejiang Provincial People’s Hospital, Hangzhou, 310014 Zhejiang China; 2 Department of Orthopedics, The Second Affiliated Hospital of Wenzhou Medical University, Xueyuan Xi Road 109#, Wenzhou, 325027 Zhejiang China; 3 Bengbu Medical College, Bengbu, 233030 Anhui China; 4 Qingdao University, Qingdao, 266071 Shandong China; 5 Operating Theater, Zhejiang Provincial People’s Hospital, Hangzhou, 310014 Zhejiang China

**Keywords:** Metal nanoparticles, Metal oxide nanoparticles, Osteoarthritis, Cartilage, Oxidative stress

## Abstract

The current understanding of osteoarthritis is developing from a mechanical disease caused by cartilage wear to a complex biological response involving inflammation, oxidative stress and other aspects. Nanoparticles are widely used in drug delivery due to its good stability in vivo and cell uptake efficiency. In addition to the above advantages, metal/metal oxide NPs, such as cerium oxide and manganese dioxide, can also simulate the activity of antioxidant enzymes and catalyze the degradation of superoxide anions and hydrogen peroxide. Degrading of metal/metal oxide nanoparticles releases metal ions, which may slow down the progression of osteoarthritis by inhibiting inflammation, promoting cartilage repair and inhibiting cartilage ossification. In present review, we focused on recent research works concerning osteoarthritis treating with metal/metal oxide nanoparticles, and introduced some potential nanoparticles that may have therapeutic effects.

## Introduction

Osteoarthritis, the most common chronic joint disease, involves the molecular, biochemical, morphological, biomechanical changes of all components in joint. It is characterized by progressive degeneration of articular cartilage and abnormal bone remodeling (Hunter and Bierma-Zeinstra 2019). Gender, obesity, genetic predisposition, mechanical load and other factors involve in the occurrence of osteoarthritis, among which aging is the most important factor (Abramoff and Caldera 2020). Systemic inflammation is an important feature of aging. Inflammatory cytokines caused chondrocytes and synoviocytes to secrete matrix metalloproteinases (MMPs) and a disintegrin and metalloproteinase with thrombospondin motifs (ADAMTs), which promoted cartilage matrix degradation. Mechanical overloading can directly promote the secretion of inflammatory cytokines by chondrocytes, and indirectly cause synovial inflammation through the generation of cartilage fragments, leading to the increase of inflammatory cytokines in synovial fluid (Scanzello and Goldring 2012). The redox state also has a significant effect on the function of chondrocytes. Reactive oxygen species (ROS) and reactive nitrogen species (RON) refer to reactive free radicals and non-free radical derivatives of oxygen and nitrogen respectively, which are the main factors affecting the redox homeostasis in cells (Liguori et al. 2018). During cellular senescence, the activity of antioxidant enzymes such as superoxide dismutase (SOD) and glutathione peroxidase (GSH) decreases, while systemic inflammation and mitochondrial dysfunction as hallmark of senescence lead to increased production of ROS/RON (Watanabe et al. 2014; Martínez et al. 2020, Coryell et al., 2021). The imbalance between the production of ROS/RON and the scavenging action of the antioxidant system results in oxidative stress. Oxidative stress causes DNA damage, protein peroxidation and lipid peroxidation. Mitochondrial DNA damage in turn aggravates mitochondrial dysfunction (Coryell et al. 2021). ROS/RON activate NF-κB pathway by modifying IκB kinase and promote the production of inflammatory cytokines (Lepetsos et al. 2019). In addition, lipid peroxides, such as ox-LDL, also promote systemic inflammation (Rhoads and Major 2018). ROS/RON inhibits cartilage matrix synthesis by regulating PI3K/Akt and MAPK pathways, and promotes cartilage matrix degradation directly or indirectly by promoting the expression of MMPs (Bai et al. 2019).

Metal/metal oxide nanoparticles (NPs) (~ 100 nm) have unique physical properties compared to microparticles (500 nm ~). NPs have a large surface area to volume ratio, which confers them high surface reactivity. On the one hand, it allows them to simulate the activity of multiple antioxidant enzymes such as SOD and catalase (CAT). In general, the smaller the particle size, the stronger the catalytic activity. On the other hand, it allows them to attach easily to cell membranes, thus increasing cell uptake (Bai et al. 2019). After entering cells, metal/metal oxide NPs exert anti-oxidative stress and anti-inflammatory effects by regulating redox state and releasing metal ions (Agarwal et al. 2019). However, high surface reactivity is also a double-edged sword. Increased cell uptake efficiency increases both bioavailability and toxicity (Mangalampalli et al., 2018, Ashrafi et al. 2019). Metal/metal oxide NPs can promote the production of ROS through mitochondrial and NADPH oxidase perturbation (Sun et al. 2018; Mukherjee and Acharya 2018). Accumulation of heavy metal ions in chondrocytes and osteoblasts causes cellular dysfunction by replacing essential elements in enzymes and disrupting the conformation of active sites, thus increasing the risk of osteoarthritis and osteoporosis (Bolduc et al., 2019, Wang et al. 2016).

Fortunately, metal/metal oxide NPs have shown strong anti-oxidative stress and the ability to promote cartilage repair at lower concentrations. This article introduces the protective metal elements for articular cartilage and the potential metal/metal oxide NPs in the treatment of osteoarthritis.

## Cellular internalization and cartilage penetration of nanoparticles

### Cellular internalization

Unlike metal ions that enter the cell via ion channels, the main way metal/metal oxide nanoparticles enter the cell is endocytosis, including clathrin-mediated endocytosis, caveolae-mediated endocytosis, lipid raft-mediated endocytosis and clathrin/caveolae/lipid-independent endocytosis (Bajpayee et al. 2015) . NPs sizes of around 2 nm require less energy to overcome the water barrier between the particle and the cell membrane, while it is easily to be retained in the hydrophobic core of the phospholipid bilayer. With the increase of particle size, the ability of NPs to escape capture is enhanced, but the energy barrier to overcome when passing through the water barrier is also significantly increased (Burgess et al. 2020). In the study of Jiang et al., gold NPs with particle size of 2–6 nm can enter cells by direct diffusion, and with the increase of particle size, the proportion of NPs entering cells by direct diffusion gradually decreases (Jiang et al. 2015). For most metal/metal oxide NPs larger than 8 nm, it is difficult for them to penetrate directly through the phospholipid bilayer or through micropores in the cell membrane to enter the cell. Metal/metal oxide NPs adhere to cell membranes through electrostatic interactions, van der Waals forces and spatial interactions. The cell membrane can be divided into a liquid ordered phase rich in lipids (lipid rafts) including cholesterol and phospholipids and a surrounding liquid-crystalline disordered phase. The adsorption of metal/metal oxide NPs causes the bending of the cell membrane. Due to the difference in thickness at the boundaries of the two lipids, metal/metal oxide NPs only need to overcome a lower energy barrier to bend the cell membrane. Therefore, they are more likely to be adsorbed at the boundaries (Ridolfi et al. 2020). Eventually, the membrane invaginates to form a vesicle that completes endocytosis. In macrophages and neutrophils, phagocytosis is also involved in the uptake of metal/metal oxide NPs.

The internalization of metal/metal oxide NPs by cells is mainly affected by the following three aspects: (i) the type and state of cells; (ii) the physicochemical properties of NPs; (iii) biological and biochemical environments. There are differences in the uptake pathways of different cells (Wathiong et al. 2019). In addition, the state of the cell also plays an important role in the uptake of metal/metal oxide NPs. Recently, Farvadi et al. found that the uptake of gold NPs by normal fibrocells (HU02) cultured on a conventional two-dimensional culture plate was significantly higher than that of cells cultured on a three-dimensional culture plate capable of mimics the in vivo cell morphology (Farvadi et al. 2018).

Among the physicochemical properties of NPs themselves, size and shape are important factors that affect the uptake of NPs. The clathrin-mediated endocytosis can form vesicles with a diameter of approximately 100–150 nm (less than 500 nm), while the caveolae-mediated endocytosis can form vesicles with a diameter of approximately 60–80 nm (Murugan et al. 2015). Wu et al. compared the uptake efficiency of B16 cells for silver NPs with particle size ranging from 5 to 100 nm, the results showed that 100 nm silver NPs had the highest uptake efficiency (Wu et al. 2019). Similarly, Ding et al. found that SMCC-7721, GES-1 and 4T1 cells had the highest uptake efficiency for gold NPs with a particle size of 80 nm within the range of 15 nm to 80 nm (Ding et al. 2018). Li et al. (2016) compared the uptake efficiency of human mesenchymal stem cells for spherical, star-shaped and rod-shaped gold NPs with size ranging from 40 to 110 nm, the results showed that spherical NPs had the highest uptake efficiency (Ding et al. 2018). In terms of endocytosis kinetics, the internalization of NPs consists of two stages: invagination and wrapping. The largest local mean curvature of NPs with various shape is different, which makes the vary degree of membrane deformation during wrapping stage. Greater deformation usually means greater resistance (Li et al. 2012). Li et al. (2016) used DPD simulation method to study the effect of the elasticity of NPs on internalization in vitro. The results showed that the soft NPs deformed when they touched the lipid membrane. On the one hand, the deformation of NPs increases the largest local mean curvature, and on the other hand, allows the NPs to bind to a large number of mobile ligands during initial contact with the lipid membrane, resulting in a decrease in the number of mobile ligands on the lipid membrane covering the other side of the NPs, thereby limiting endocytosis (Li et al. 2015). However, the effect of elasticity on internalization of metal/metal oxide NPs with high density is negligible.

Surface potential and surface properties of NPs also play an important role in their internalization process. Because cell membranes usually carry negative charges, cationic NPs are more likely to attach to cells, while anionic NPs have ability to circulate steadily in the body. Surface modification of metal/metal oxide NPs can effectively reduce aggregation. The agglomeration of NPs increases the size and accelerates the deposition. In addition, the uptake of NPs can also be increased by binding specific or non-specific ligands on the surface of NPs (Li et al. 2015).

Biological and biochemical environments can influence the uptake of NPs too. After the metal/metal oxide NPs enter the biological fluid, they are quickly covered with organic materials, such as lipids and carbonic acid, thus forming a corona. Proteins play a main role in corona formation (Pareek et al. 2018). The factors affecting the formation of protein corona include particle size, shape, surface area to volume ratio and surface charge. On the one hand, protein corona influences the biological behavior of NPs. Protein corona can be recognized as ligands by receptors on the cell membrane, thus facilitating the uptake of NPs (Binnemars-Postma et al. 2016). Ding et al. compared the uptake of gold NPs by 7721 cells in serum and serum-free environments, the results showed that the uptake of silver NPs by 7721 cells in serum-free environments was changed to clathrin and caveolin-independent pathways (Ding et al. 2018). On the other hand, the NPs might also cause the proteins in the protein corona to unfold, thus causing biological toxicity.

### Cartilage penetration

Articular cartilage is composed of chondrocytes and extracellular matrix. Glycosaminoglycans and proteoglycans fill a fibrous network of collagen and elastin to form the extracellular matrix. In terms of biomechanical behavior, articular cartilage consists of liquid phase composed mainly of water and solid phase represented by collagen and proteoglycan. The flow of the liquid phase in the solid phase provides the elasticity of the cartilage (Carballo et al. 2017). Repeated loading causes the decrease of tensile strength, stiffness and thickness of articular cartilage, leading to the occurrence of osteoarthritis (Vazquez et al. 2019). The fibrous network of collagen and elastin has a hydration spacing of approximately 0–100 nm, while the gap between the glycosaminoglycan and proteoglycan side chains filled in them is about 0–15 nm (Krishnan and Grodzinsky 2018; Torzilli et al. 1997). The permeability of metal/metal oxide nanoparticles in cartilage mainly depends on particle size and surface potential. The metal/metal oxide NPs with particle size less than 15 nm have good permeability in cartilage (Bajpayee et al. 2015). In addition, since cartilage tissue carries a negative charge, metal/metal NPs with a positive electric potential are more likely to penetrate into cartilage. High cartilaginous permeability not only means higher utilization, but also means longer endochondral retention time. Metal/metal oxide NPs are usually able to remain in the joint cavity for a longer period of time, which can be regarded as a ‘sustained-release agent’ for metal ions to some extent.

## Nanoparticles that have shown cartilage protective effects

### Gold and gold nanoparticles

#### Therapeutic mechanism of gold nanoparticles in osteoarthritis

In past studies, gold NPs have shown good biocompatibility to most tissues in the human body (Cabuzu et al. 2015). Pascarelli et al. evaluated the toxicity of gold NPs with particle size of 50 nm to human osteoarthritis chondrocytes at different concentrations. They treated chondrocytes with gold NPs of 20 μM, 40 μM, 80 μM, 160 μM, and 250 μM for 24 h. The results showed that chondrocyte viability was significantly inhibited (less than 40%) at concentrations above 160 μM. The expression of inducible nitric oxide synthase (iNOS) and chondrolysis-related proteins, including MMP-1/3/13 and ADAMTs-4/5, was increased (Pascarelli et al. 2013). In another study evaluating the cytotoxicity of gold NPs of different sizes to rabbit chondrocytes, only gold NPs at 13 nm significantly induced mitochondrial dysfunction and apoptosis compared with gold NPs at 3 nm and 45 nm (Huang et al. 2016). These results show that gold NPs also have good biocompatibility with cartilage.

Gold NPs have a good ability to resist oxidative stress. After treating RAW264.7 macrophages with poly-N-vinylpyrrolidone modified gold NPs (50 nm), Kingston et al. found that gold NPs of 5, 25 and 50 μg/ml significantly reduced ROS production, and the inhibition effect was strongest at 25 μg/ml (Kingston et al. 2016). In another study of methamphetamine-induced liver injury treated with gold NPs (7.4 ± 1.6 nm), gold NPs significantly reduced myeloperoxidase (MPO) activity, increased glutathione peroxidases (GPX) activity, and significantly reduced malondialdehyde production (de Carvalho et al. 2018). MPO is mainly expressed in neutrophils and monocytes and can catalyze the synthesis of hypochlorous acid. The decrease of MPO activity means the reduction of oxidative stress (Ndrepepa 2019). In addition to regulating the expression levels of oxidase and antioxidant enzyme, gold NPs themselves can also simulate the activities of various antioxidant enzymes such as GSH, SOD and peroxidase, and catalyze the decomposition of ROS (Dashtestani et al. 2019).

Khan et al. discussed the mechanism by which gold NPs inhibit inflammation. They used gold NPs, about 15 nm in size, to intraperitoneally inject a mouse model of collagen-induced arthritis once a week at a dose of 20 μg/kg. They collected serum after 20 days and found that the expressions of NF-kB, cyclooxygenase-2 (COX-2), TNF-α, and IL-1β in the serum of the mice treated with gold NPs were significantly reduced compared with the negative control group. This suggests that gold NPs can inhibit inflammation by inhibiting the NF-kB pathway (Khan and Khan 2018). Synovitis plays an important role in the progression of osteoarthritis. Chondrodegradation products stimulate synovial fibroblasts and synovial macrophages to secrete cytokines such as IL-1β and TNF-α, and promote chondrocyte secrete proteases such as MMP-13 and ADAMTs-5 (Mathiessen and Conaghan 2017). Park et al. synthesized triamcinolone-gold NPs using gold NPs with a particle size of 20 nm and triam. The results showed that IL-1β, IL-6, TNF-α, MMP-1 and MMP-3 were down-regulated in a dose-dependent manner. While only triamcinolone-gold NPs significantly increased the expression of IL-4, Arg-1 and IL-10. This suggests that M1-type macrophages were transformed into M2-type macrophages under the treatment of triamcinolone-gold NPs (Park et al. 2020).

**Fig. 1:**
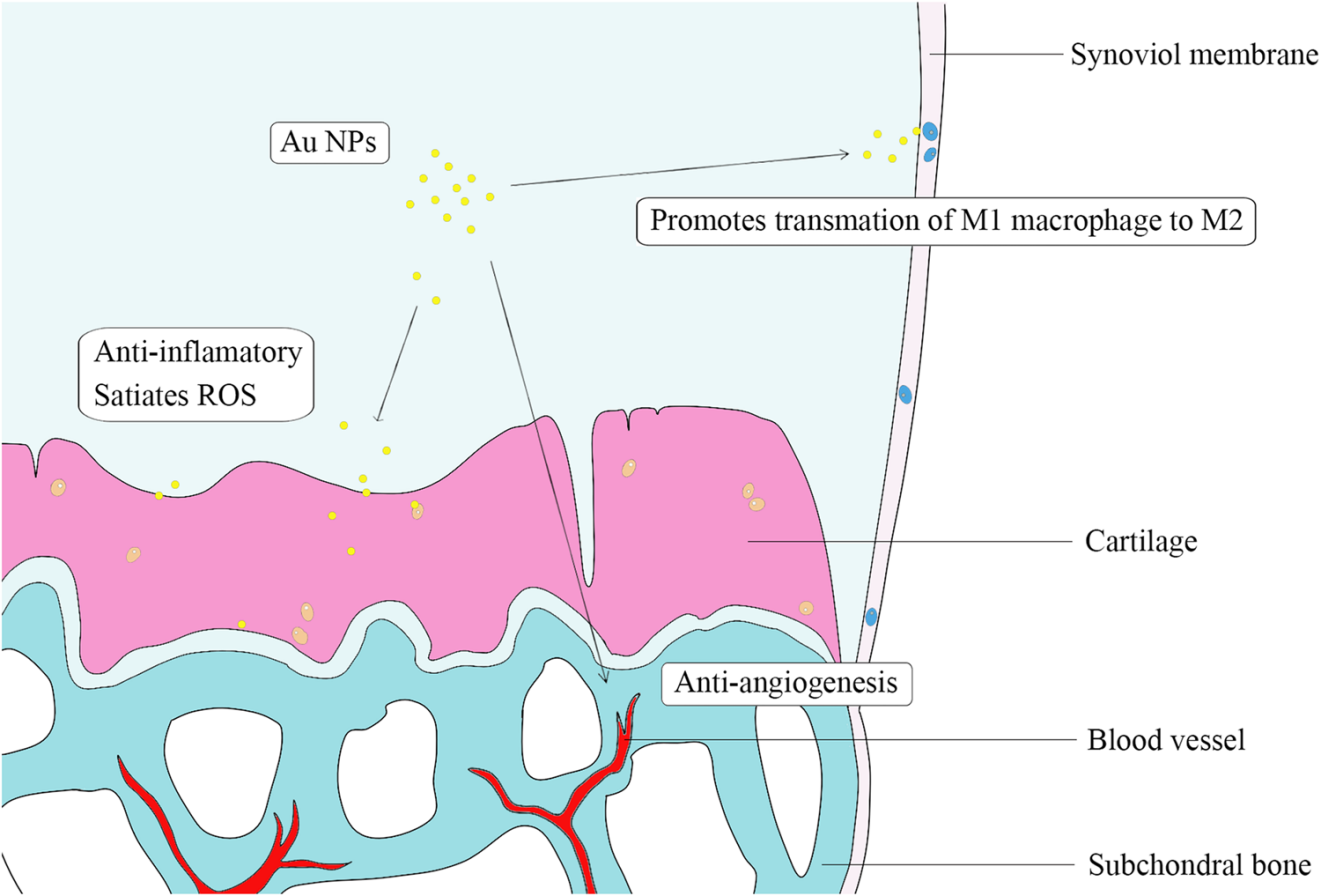
Mechanism of gold nanoparticles in the treatment of osteoarthritis. After being ingested by chondrocytes and synovial cells, gold nanoparticles promote the decomposition of intracellular reactive oxygen species, inhibit the expression of inflammation-related genes, and inhibit the expression of angiogenesis related genes. Gold nanoparticles promote the transformation of pro-inflammatory M1-type macrophages to anti-inflammatory M2-type macrophages

Although angiogenesis plays an important role in early cartilage development, vascular structure is not expressed in mature cartilage (Lingaraj et al. 2010, Zelzer et al. 2004). In osteoarthritis, the imbalance between angiogenesis and inhibitory factors leads to the advantage of angiogenesis, increases angiogenesis in synovium and meniscus, deep layer ossification of articular cartilage and osteophyte formation, which promotes cartilage destruction. Several past studies have shown that gold NPs have excellent antiangiogenic abilities (Darweesh et al. 2019) (Fig. 1).

However, gold NPs are still potentially dangerous. Zhang et al. (2019) treated mouse osteoblasts with 20 nm and 40 nm gold NPs at the concentration of 0.1 nM and 1 nM, respectively. The results showed that alkaline phosphatase (ALP) activity of osteoblasts was increased and the number of calcium nodules was increased. The expressions of osteogenic specific genes Runx-2, BMP-2 and osteocalcin (OCN) were significantly increased. Phosphorylated Erk protein was increased. These results suggest that gold NPs promote osteogenesis of osteoblasts through the Erk pathway (Zhang et al. 2014). This may aggravate osteophyte formation in patients with osteoarthritis.

#### Progress of gold nanoparticles in the treatment of osteoarthritis

In recent years, therapeutic agents related to gold NPs have been continuously developed. On the one hand, the way in which gold NPs can be delivered as therapeutic agents continues to improve. For example, using liposomes to enclose gold NPs absorbed with fish oil, which increase the stability (Sarkar et al. 2019). On the other hand, a growing number of drugs are being transported using gold NPs for their good biocompatibility and drug binding ability, such as hyaluronic acid, chondroitin sulfate, 2-[(aminocarbonyl)amino]-5-(4-fluorophenyl)-3-thiophenecarboxamide (TPCA-1), and rutin (Dwivedi et al. 2015, Sansanaphongpricha et al. 2020, Gul et al. 2018, Wang et al. 2020).

Gold NPs have been widely used in the photothermal therapy of tumors due to their excellent photothermal conversion efficiency (Dwivedi et al. 2015, Sansanaphongpricha et al. 2020, Gul et al. 2018, Wang et al. 2020). Lee et al. designed poly(DL-lactic-co-glycolic acid) Au half-shell NPs carrying methotrexate (MTX) and binding arginylglycylaspartic acid peptides on the surface for targeting inflammatory synovium. Under the action of near-infrared resonance, MTX-PLGA-gold NPs realize photothermal controlled drug delivery (Lee et al. 2013). In summary, the potential of gold NPs in the treatment of osteoarthritis is constantly being explored.

### Manganese and manganese oxide nanoparticles

#### Therapeutic mechanism of manganese oxide nanoparticles in osteoarthritis

Manganese, as a essential nutrient, is involved in various intracellular functions, such as the maintenance of manganese SOD (MnSOD) activity in the form of cofactor. Excess manganese accumulates in various tissues of the body, including the liver, bone and brain, the latter being the main target of manganese toxicity (Chen et al. 2018). Excess manganese ions can cause mitochondrial dysfunction by interfering with calcium homeostasis, and in addition, can increase the production of ROS (Tuschl et al. 2013).

Manganese oxide NPs play a dual role in oxidative stress. Alarifi et al. found that the particle size of 50 nm manganese tetroxide NPs significantly increased the production of ROS and inhibited the activities of SOD and GPX enzyme in SH-SY5Y neuroblastoma cells at the concentration of 30 μg/ml (Alarifi et al. 2017). Similarly, manganese tetroxide NPs with a particle size of about 20 nm significantly increased the production of ROS and apoptosis in PC12 neurons at a concentration of 5 μg/ml (Chen et al. 2020). After intraperitoneal injection of 30–60 nm manganese dioxide nanoparticles at a dose of 50 ug/kg in rats for 15 days, the production of ROS and apoptosis in hippocampal nerve cells were increased (Sadeghi et al. 2018).

These results suggest that manganese oxide NPs can induce the production of ROS to a certain extent. On the other hand, manganese oxide NPs can also play a role in anti-oxidative stress by simulating the activities of peroxidase and SOD (Tootoonchi et al. 2017). Singh et al. (2018) found that manganese tetroxide nanoparticles with a diameter of about 50 nm significantly reduced the production of ROS in HEK293T kidney cells and HeLa cells induced by H_2_O_2_. The accumulation of protein carbonyl, DNA double strand breakage and increased activity of apoptosis-related protein caspase-3/7 caused by ROS was also alleviated. Besides, the endogenous antioxidant enzyme system was not affected, including peroxiredoxin, CAT, SOD and glutathione (Singh et al. 2019). Similarly, Kuthati et al. pretreated bone marrow-derived macrophages with manganese oxide NPs with a particle size of 10 nm, after which the production of ROS induced by PAM3CSK4 was significantly reduced (Kuthati et al. 2019). Manganese oxide NPs also reduced the expression of inflammation-related proteins, such as COX-2.

#### Therapeutic effect of manganese oxide nanoparticles in osteoarthritis

Polyethylene glycol (PEG) is a degradable polymer made up of repeated ethylene glycol units [-(CH_2_CH_2_O)_n_], which can effectively reduce aggregation as a coating of NPs. The modification of low molecular weight PEG helped to maintain a smaller particle size, while the modification of high molecular weight PEG made the NPs more stable in vivo (Morgenstern et al. 2017; Abelha et al. 2019). Recently, Kumar et al. using potassium permanganate as manganese source and poly(allyl amine) hydrochloride (PAH) as reducing agent, prepared manganese dioxide NPs with particle size of 12.85 nm, and increased its stability by conjugating with PEG (Kumar et al. 2019). PEG can bind to manganese dioxide nanoparticles in the form of coordination bonds. In addition, it can covalently bind with the NH_2_ group on PAH adsorbed on the surface of the manganese dioxide NPs (D'Souza and Shegokar 2016). They evaluated the efficacy of the NPs in the treatment of osteoarthritis. The results showed that PEG-MnO_2_ NPs showed no cytotoxicity to mouse chondrocytes, synovial cells, mesenchymal stem cells and bone marrow derived macrophages in the range of 0–100 µg/mL. After treatment of 5 µg/mL PEG-MnO_2_ NPs, cartilage degradation products glucan aminoglycans and NO production were significantly reduced in cartilage grafts stimulated by IL-1β. The expression of nitric oxide synthase, MMP-1, MMP-13 and ADAMTs5 were significantly decreased after treatment with 5 g/ml PEG-MnO 2 NPs in chondrocyte stimulated by IL-1β. The expression of anti-oxidative stress related genes, including GPX, MnSOD, extracellular SOD, CAT and Nrf2 pathway related genes, returned to normal levels. In vivo tests indicated that PEG-MnO_2_ NPs remained in the articular cavity for more than 7 days without long-term adverse effects.

### Cerium and cerium oxide nanoparticles

#### Therapeutic mechanism of cerium oxide nanoparticles

Because the liver is the main accumulation organ of NPs in the body, therapeutic studies based on the anti-oxidative stress effect of cerium oxide NPs are mainly focused on liver at present (Casals et al. 2020). Cerium oxide NPs also mimic the catalytic activity of antioxidant enzymes such as SOD and CAT. Adebayo et al. 2020 injected cerium NPs with particle size less than 10 nm into rats intraperitoneally at a dose of 100 μg/kg day and 200 μg/kg day for 8 days continuously, then intraperitoneal injection of diethylnitrosamine induces liver injury. The results showed that cerium oxide NPs significantly reduced the production of NO and lipid peroxides induced by diethylnitrosamine, decreased the activities of MPO and restored the activities of CAT activities and GPX. The apoptotic protein Bcl-2 was significantly reduced (Casals et al. 2020). Similarly, cerium oxide NPs alleviated oxidative damage induced by H_2_O_2_ and LPS in HepG2 hepatoma cells (4.6 ± 2 nm;10 μg/ml in vitro) (Carvajal et al. 2019). In a mouse model of cirrhosis, cerium oxide NPs reduced oxidative stress in portal vein endothelial cells in vitro and in vivo (4–20 nm;1 μg/ml in vitro; 0.1 mg/kg in vivo, i.t.) (Ribera et al. 2019).

The anti-oxidative stress ability of cerium oxide NPs may be derived from the conversion to Ce^3+^ and Ce^4+^. The conversion to Ce^3+^ to Ce^4+^ makes the cerium oxide NPs exhibit SOD-like activity, while the conversion to Ce^4+^ to Ce^3+^ makes the cerium oxide NPs exhibit CAT-like activity (Zheng et al. 2019).

#### Therapeutic effect of cerium oxide nanoparticles in osteoarthritis

Recently, Lin et al. (2020) evaluated the role of cerium oxide NPs with 30–60 nm size in osteoarthritis. The results showed that the cerium oxide NPs showed no cytotoxicity in the range of 0–0.02 μg/ml. Compared with 0.01 μg/mL cerium oxide NPs alone or 0.1% hyaluronic acid group, cerium oxide NPs combined with hyaluronic acid group significantly reduced the apoptosis of chondrocytes induced by H_2_O_2_, increased the expression of aggrecan (ACAN), Col1α and Col2α1, and increased the accumulation of sulfated proteoglycans (Zheng et al. 2019).

Notably, compared with other cells, cerium oxide NPs have been shown to inhibit chondrocyte activity at low concentrations, and its therapeutic effect in osteoarthritis needs to be further evaluated.

## Nanoparticles with the potential for cartilage protection

### Magnesium and magnesium oxide nanoparticles

#### Effect of magnesium ions on osteoarthritis

Magnesium is closely related to the regulation of cartilage function. High serum magnesium was inversely associated with radiographic severity of osteoarthritis (Zeng et al. 2015). Increased magnesium intake increases the thickness and volume of knee cartilage (Veronese et al. 2019). In patients with osteoarthritis, decreased magnesium intake was associated with decreased joint function and increased pain (Shmagel et al. 2018).

Magnesium ions play a protective role in osteoarthritis in several ways. Yao et al. cultured human osteoarthritis cartilage grafts with 20 mmol/L MgCI_2_, and found that the production of MMP-13, ADAMTs-5, IL-6 and IL-1β decreased, while the expression of COL2α1 and ACAN increased. Intraarticular injection of 0.5 mol/L MgCI_2_ also showed cartilage protective and antisynovitis effects in a mouse model of osteoarthritis (Yao et al. 2019). These evidence suggest that magnesium ions can inhibit inflammation, reduce cartilage degradation and promote cartilage synthesis in osteoarthritis. In another study, magnesium ions suppressed the expression of osteogenic specific genes ALP, Runx2, and COL10α1, and increased the expression of SOX9. In this process, the phosphorylation of Erk protein was inhibited, which inhibited the activation of Erk pathway (Yue et al. 2019). This suggests that magnesium ions inhibits the hypertrophic ossification of chondrocytes. In addition, the presence of magnesium ions can also inhibit autophagy of chondrocytes.

#### Therapeutic effect of magnesium oxide nanoparticles

At present, there are few studies related to the therapeutic effect of magnesium oxide NPs. Nodeh et al. evaluated the effects of dispersed magnesium oxide NPs with a size of about 100 nm on islet cells. The results showed that the levels of ROS and lipid peroxidation were significantly decreased under the treatment of 10 and 100 μg/ml magnesium oxide NPs. One hundred microgram/ml magnesium oxide NPs significantly increased the cell viability, decreased the expression of caspase-3/9, and inhibited the apoptosis of islet cells (Moeini-Nodeh et al. 2017). Magnesium oxide NPs have the ability to inhibit oxidative stress.

**Fig. 2:**
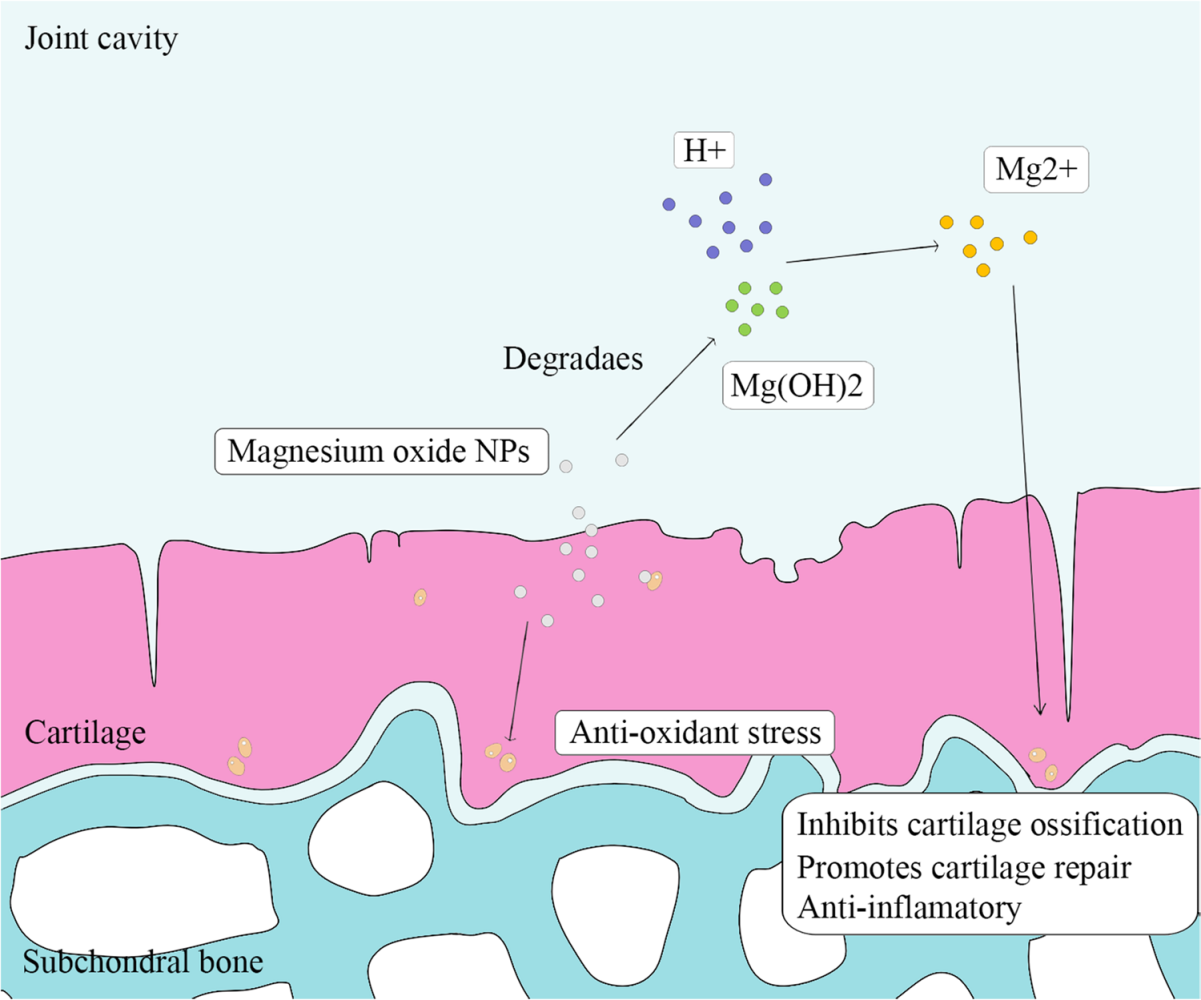
Potential mechanism of magnesium oxide nanoparticles in the treatment of osteoarthritis. Magnesium oxide nanoparticles promote scavenging of reactive oxygen species in cells. Magnesium oxide nanoparticles are degraded to Mg(OH)_2_ in the synovial fluid, which releases magnesium ions while neutralizing the acidic environment in the joint cavity, promotes the synthesis of cartilage matrix, and inhibits the expression of osteogenic specific genes and inflammation-related genes

Compared with normal joint fluid, joint fluid in patients with osteoarthritis is more acidic. Magnesium oxide NPs was degraded to Mg(OH)_2_ and further converted to MgCI_2_ (Suryavanshi et al. 2017)_._ In this process, magnesium oxide NPs may also play a role in improving the pH of the joint fluid while producing biologically active magnesium ions. In conclusion, magnesium oxide NPs have certain potential for the treatment of osteoarthritis (Fig. 2).

### Zinc and zinc oxide nanoparticles

#### Effect of zinc ions on osteoarthritis

Zinc ions may play a dual role in the progression of osteoarthritis. According to studies, high serum zinc is associated with a reduced risk of cartilage calcification (He et al. 2020). However, there is also a study which finds an inherited high zinc status increased the risk of osteoarthritis (Zhou et al. 2021).

As a cofactor of matrix metalloproteinase and thrombin proteinase, zinc plays an important role in cartilage destruction (Malemud 2019). The expression of Zn2^+^ transporter ZIP8 is increased in osteoarthritis cartilage. The increase in ZIP8 expression results in the increase in intracellular Zn2^+^ content, and activates downstream transcription factor MTF1, which upregulates the expression of matrix-degrading enzymes, such as MMP-3 and MMP-13 (Kim et al. 2014). On the other hand, zinc ions have a protective effect on cartilage under oxidative stress. As reported by Huang et al., zinc ions reversed the decrease in the expression of energy metabolism-related proteins (including glucose transporter-1, hexokinase-2, pyruvate dehydrogenase E1 component subunit alpha) and autophagy related proteins (including ATG5/7, LC3-II, PINK1) induced by iodoacetic acid in SW1353 osteosarcoma cells (Huang et al. 2020, 2018). The decrease of GPX1 and Mn-SOD expression was saved, while increase of IL-1β and MMP-13 expression of was inhibited. In addition, Nrf2 and its upstream kinase phosphorylated Akt were increased. Nrf2 plays an important protective role in the progression of osteoarthritis by inhibiting oxidative stress (Loeser et al. 2016). In another study, adding zinc ions to the drinking water of iodoacetic acid-induced osteoarthritis mice reduced cartilage damage (Huang et al. 2018).

#### Therapeutic effect of zinc oxide nanoparticles

Zinc oxide NPs also have anti-oxidative stress, anti-inflammatory and other effects. The activities of SOD, peroxidase and CAT in liver of gout mice were recovered after oral administration of zinc oxide NPs with a diameter of 37 nm. The reaction products of ROS and thiobarbituric acid reactive substances were reduced too (Kiyani et al. 2019). On the anti-inflammatory effect, zinc oxide NPs with particle size of 200 significantly inhibited the NF-kB pathway in the range of 0–10 μg/mL, and reduced the expression of COX-2 and iNOS (Kim and Jeong 2015). The study of Khader and Arinzeh (2020) suggested the effect of zinc oxide NPs on chondrocytes (Kim and Jeong 2015). Polycaprolactone (PCL) is an aliphatic polyester made up of repeated hexanoate units that are widely used in the long term delivery of drugs. Khader and Arinzeh (2020) dissolved 80,000 molecular weight of polycaprolactone (PCL) [-(C_6_H_10_O_2_)_n_] and zinc oxide NPs with an average particle size of 63 nm in an organic solvent, and synthesized a composite scaffold capable of sustainably releasing zinc oxide NPs by electrospinning. Then the scaffold was inserted into the cell plate in the form of a disc, and human mesenchymal stem cells were seeded on it. The results showed that cells growing on scaffolds containing 1% or 2.5% zinc oxide NPs expressed higher levels of SOX9, ACAN and Col2 after 7, 14, and 28 days of culture in cartilage medium. After 28 days of culture in growth medium, ALP activity and hydroxyproline production were higher in cells grown on scaffolds containing 5% or 10% zinc oxide NPs. The expression of the osteogenic genes Runx-2, OCN, Osterix, and VEGF in cells grown on scaffolds containing 1% or 2.5% zinc oxide NPs was similar to that in the control group.

## Conclusion

In recent years, the green synthesis of metal/metal oxide NPs has attracted more and more attention. Ahn et al. synthesized gold NPs with skate (*Dipturus chilensis*) cartilage waste extract as a green reducing agent (Ahn et al. 2018). Ogunyemi et al. synthesized magnesium oxide and manganese dioxide NPs with chamomile extracts (Ogunyemi et al. 2019). NPs made from bacteria and fungi or made through plant extract are safer than traditional chemical synthesis methods, reducing the use of toxic chemicals. Meanwhile, the green synthesis process is usually simpler and uses less energy (Singh et al. 2016). Compared with expensive chemicals, the use of green plant extracts for synthesis is also more cost-effective.

Nanotechnology holds broad promise in the treatment of osteoarthritis. Compared with drugs, NPs are more stable in the body and have a longer half-life, so they are often used as carriers to deliver drugs. Meanwhile, NPs are also excellent sustained-release systems, which can continuously and stably release the loaded drugs. NPs have a long retention time in the joint cavity (Kumar et al. 2019; Liu et al. 2019). Cartilage damage and synovial inflammation play an important role in the progression of osteoarthritis. Type II collagen is the main component of cartilage matrix, and by binding antibodies for type II collagen to the surface of NPs, the targeted uptake of NPs by damaged articular cartilage can be promoted (Noyori et al. 1994). The increased proportion of angiogenic endothelial cells in the inflammatory synovium increases the expression of αvβ3 integrins. Modification of specific ligand such as vitronectin enables NPs to target the inflammatory synovium (Zhou et al. 2009). Systemic administration that does not intrude into the joint space, such as oral or intravenous administration, has the advantage of reducing discomfort and the risk of joint infection. Targeted modification of NPs makes systemic drug delivery more promising, reducing the dose needed to trigger therapeutic effects while reducing the side effects of untargeted absorption. Metal/metal oxide NPs have the above advantages, but also can simulate the activity of antioxidant, thus exerting the role of anti-oxidative stress. In addition, metal/metal oxide NPs also have good antimicrobial activity, which makes them potentially promising for treatment of septic arthritis. The biocompatibility of NPs is greatly affected by the degradability of NPs and the difficulty of removing the degradation products. Metal/metal oxide NPs and their degradation release metal ions are easy to accumulate in vivo, which may lead to potential toxicity. Sun et al. found that the ability of metal nanoparticles to induce the production of reactive oxygen species was positively correlated with the amount of positive charge carried by nanoparticles and the degree of hydrophobicity (Sun et al. 2018). Although metal/metal oxide NPs have not shown significant toxicity at therapeutic doses in the literature reviewed here, their long-term safety needs to be further monitored. In summary, in this article, we summarize the current status of metal/metal oxide NPs in the treatment of osteoarthritis and propose some promising NPs.

## Data Availability

Not applicable.

## References

[CR1] Abelha TF, Neumann PR, Holthof J, Dreiss CA, Alexander C, Green M, Dailey LA (2019). Low molecular weight PEG-PLGA polymers provide a superior matrix for conjugated polymer nanoparticles in terms of physicochemical properties, biocompatibility and optical/photoacoustic performance. J Mater Chem B.

[CR2] Abramoff B, Caldera FE (2020). Osteoarthritis: pathology, diagnosis, and treatment options. Med Clin North Am.

[CR3] Adebayo OA, Akinloye O, Adaramoye OA (2020). Cerium oxide nanoparticles attenuate oxidative stress and inflammation in the liver of diethylnitrosamine-treated mice. Biol Trace Elem Res.

[CR4] Agarwal H, Nakara A, Shanmugam VK (2019). Anti-inflammatory mechanism of various metal and metal oxide nanoparticles synthesized using plant extracts: a review. Biomed Pharmacother.

[CR5] Ahn EY, Lee YJ, Choi SY, Im AR, Kim YS, Park Y (2018). Highly stable gold nanoparticles green-synthesized by upcycling cartilage waste extract from yellow-nose skate (Dipturus chilensis) and evaluation of its cytotoxicity, haemocompatibility and antioxidant activity. Artif Cells Nanomed Biotechnol.

[CR6] Alarifi S, Ali D, Alkahtani S (2017). Oxidative stress-induced DNA damage by manganese dioxide nanoparticles in human neuronal cells. Biomed Res Int.

[CR7] Ashrafi HA, Naserzadeh P, Mortazavian AM, Mehravi B, Ashtari K, Seydi E, Salimi A (2019). Comparison of the effects of MnO(2)-NPs and MnO(2)-MPs on mitochondrial complexes in different organs. Toxicol Mech Methods.

[CR8] Bai Y, Gong X, Dou C, Cao Z, Dong S (2019). Redox control of chondrocyte differentiation and chondrogenesis. Free Radic Biol Med.

[CR9] Bajpayee AG, Scheu M, Grodzinsky AJ, Porter RM (2015). A rabbit model demonstrates the influence of cartilage thickness on intra-articular drug delivery and retention within cartilage. J Orthop Res.

[CR10] Binnemars-Postma KA, Ten HH, Storm G, Prakash J (2016). Differential uptake of nanoparticles by human M1 and M2 polarized macrophages: protein corona as a critical determinant. Nanomedicine (Lond).

[CR11] Bolduc JA, Collins JA, Loeser RF (2019). Reactive oxygen species, aging and articular cartilage homeostasis. Free Radic Biol Med.

[CR12] Burgess S, Wang Z, Vishnyakov A, Neimark AV (2020). Adhesion, intake, and release of nanoparticles by lipid bilayers. J Colloid Interface Sci.

[CR13] Cabuzu D, Cirja A, Puiu R, Grumezescu AM (2015). Biomedical applications of gold nanoparticles. Curr Top Med Chem.

[CR14] Carballo CB, Nakagawa Y, Sekiya I, Rodeo SA (2017). Basic science of articular cartilage. Clin Sports Med.

[CR15] Carvajal S, Perramón M, Casals G, Oró D, Ribera J, Morales-Ruiz M, Casals E, Casado P, Melgar-Lesmes P, Fernández-Varo G, Cutillas P, Puntes V, Jiménez W (2019). Cerium oxide nanoparticles protect against oxidant injury and interfere with oxidative mediated kinase signaling in human-derived hepatocytes. Int J Mol Sci.

[CR16] Casals E, Zeng M, Parra-Robert M, Fernández-Varo G, Morales-Ruiz M, Jiménez W, Puntes V, Casals G (2020). Cerium oxide nanoparticles: advances in biodistribution, toxicity, and preclinical exploration. Small.

[CR17] Chen P, Bornhorst J, Aschner M (2018). Manganese metabolism in humans. Front Biosci (landmark Ed).

[CR18] Chen X, Wu G, Zhang Z, Ma X, Liu L (2020). Neurotoxicity of Mn(3)O(4) nanoparticles: apoptosis and dopaminergic neurons damage pathway. Ecotoxicol Environ Saf.

[CR19] Coryell PR, Diekman BO, Loeser RF (2021). Mechanisms and therapeutic implications of cellular senescence in osteoarthritis. Nat Rev Rheumatol.

[CR20] Darweesh RS, Ayoub NM, Nazzal S (2019). Gold nanoparticles and angiogenesis: molecular mechanisms and biomedical applications. Int J Nanomedicine.

[CR21] Dashtestani F, Ghourchian H, Najafi A (2019). Silver-gold-apoferritin nanozyme for suppressing oxidative stress during cryopreservation. Mater Sci Eng C Mater Biol Appl.

[CR22] de Carvalho TG, Garcia VB, de Araújo AA, Da SGL, Silva H, Guerra G, de Castro ME, de Carvalho LR, Da SCD, Cruz LJ, Chan AB, de Araújo JR (2018). Spherical neutral gold nanoparticles improve anti-inflammatory response, oxidative stress and fibrosis in alcohol-methamphetamine-induced liver injury in rats. Int J Pharm.

[CR23] Ding L, Yao C, Yin X, Li C, Huang Y, Wu M, Wang B, Guo X, Wang Y, Wu M (2018). Size, shape, and protein corona determine cellular uptake and removal mechanisms of gold nanoparticles. Small.

[CR24] D’Souza AA, Shegokar R (2016). Polyethylene glycol (PEG): a versatile polymer for pharmaceutical applications. Expert Opin Drug Deliv.

[CR25] Dwivedi P, Nayak V, Kowshik M (2015). Role of gold nanoparticles as drug delivery vehicles for chondroitin sulfate in the treatment of osteoarthritis. Biotechnol Prog.

[CR26] Farvadi F, Ghahremani MH, Hashemi F, Reza HM, Raoufi M, Zanganeh S, Atyabi F, Dinarvand R, Mahmoudi M (2018). Cell shape affects nanoparticle uptake and toxicity: an overlooked factor at the nanobio interfaces. J Colloid Interface Sci.

[CR27] Gul A, Kunwar B, Mazhar M, Faizi S, Ahmed D, Shah MR, Simjee SU (2018). Rutin and rutin-conjugated gold nanoparticles ameliorate collagen-induced arthritis in rats through inhibition of NF-κB and iNOS activation. Int Immunopharmacol.

[CR28] He H, Wang Y, Yang Z, Ding X, Yang T, Lei G, Li H, Xie D (2020). Association between serum zinc and copper concentrations and copper/zinc ratio with the prevalence of knee chondrocalcinosis: a cross-sectional study. BMC Musculoskelet Disord.

[CR29] Huang H, Quan YY, Wang XP, Chen TS (2016). Gold nanoparticles of diameter 13 nm induce apoptosis in rabbit articular chondrocytes. Nanoscale Res Lett.

[CR30] Huang LW, Huang TC, Hu YC, Hsieh BS, Chiu PR, Cheng HL, Chang KL (2020). Zinc protects chondrocytes from monosodium iodoacetate-induced damage by enhancing ATP and mitophagy. Biochem Biophys Res Commun.

[CR31] Huang TC, Chang WT, Hu YC, Hsieh BS, Cheng HL, Yen JH, Chiu PR, Chang KL (2018). Zinc protects articular chondrocytes through Changes in Nrf2-mediated antioxidants, cytokines and matrix metalloproteinases. Nutrients.

[CR32] Hunter DJ, Bierma-Zeinstra S (2019). Osteoarthritis. Lancet.

[CR33] Jiang Y, Huo S, Mizuhara T, Das R, Lee YW, Hou S, Moyano DF, Duncan B, Liang XJ, Rotello VM (2015). The interplay of size and surface functionality on the cellular uptake of sub-10 nm gold nanoparticles. ACS Nano.

[CR34] Khader A, Arinzeh TL (2020). Biodegradable zinc oxide composite scaffolds promote osteochondral differentiation of mesenchymal stem cells. Biotechnol Bioeng.

[CR35] Khan MA, Khan MJ (2018). Nano-gold displayed anti-inflammatory property via NF-kB pathways by suppressing COX-2 activity. Artif Cells Nanomed Biotechnol.

[CR36] Kim JH, Jeon J, Shin M, Won Y, Lee M, Kwak JS, Lee G, Rhee J, Ryu JH, Chun CH, Chun JS (2014). Regulation of the catabolic cascade in osteoarthritis by the zinc-ZIP8-MTF1 axis. Cell.

[CR37] Kim MH, Jeong HJ (2015). Zinc oxide nanoparticles suppress LPS-induced NF-κB activation by inducing A20, a negative regulator of NF-κB, in RAW 264.7 macrophages. J Nanosci Nanotechnol.

[CR38] Kingston M, Pfau JC, Gilmer J, Brey R (2016). Selective inhibitory effects of 50-nm gold nanoparticles on mouse macrophage and spleen cells. J Immunotoxicol.

[CR39] Kiyani MM, Butt MA, Rehman H, Ali H, Hussain SA, Obaid S, Arif HM, Mahmood T, Bokhari S (2019). Antioxidant and anti-gout effects of orally administered zinc oxide nanoparticles in gouty mice. J Trace Elem Med Biol.

[CR40] Krishnan Y, Grodzinsky AJ (2018). Cartilage diseases. Matrix Biol.

[CR41] Kumar S, Adjei IM, Brown SB, Liseth O, Sharma B (2019). Manganese dioxide nanoparticles protect cartilage from inflammation-induced oxidative stress. Biomaterials.

[CR42] Kuthati Y, Busa P, Goutham DV, Wong CS (2019). Manganese oxide nanozymes ameliorate mechanical allodynia in a rat model of partial sciatic nerve-transection induced neuropathic pain. Int J Nanomedicine.

[CR43] Lee SM, Kim HJ, Ha YJ, Park YN, Lee SK, Park YB, Yoo KH (2013). Targeted chemo-photothermal treatments of rheumatoid arthritis using gold half-shell multifunctional nanoparticles. ACS Nano.

[CR44] Lepetsos P, Papavassiliou KA, Papavassiliou AG (2019). Redox and NF-κB signaling in osteoarthritis. Free Radic Biol Med.

[CR45] Li J, Li JJ, Zhang J, Wang X, Kawazoe N, Chen G (2016). Gold nanoparticle size and shape influence on osteogenesis of mesenchymal stem cells. Nanoscale.

[CR46] Li Y, Yue T, Yang K, Zhang X (2012). Molecular modeling of the relationship between nanoparticle shape anisotropy and endocytosis kinetics. Biomaterials.

[CR47] Li Y, Zhang X, Cao D (2015). Nanoparticle hardness controls the internalization pathway for drug delivery. Nanoscale.

[CR48] Liguori I, Russo G, Curcio F, Bulli G, Aran L, Della-Morte D, Gargiulo G, Testa G, Cacciatore F, Bonaduce D, Abete P (2018). Oxidative stress, aging, and diseases. Clin Interv Aging.

[CR49] Lin YW, Fang CH, Meng FQ, Ke CJ, Lin FH (2020). Hyaluronic acid loaded with cerium oxide nanoparticles as antioxidant in hydrogen peroxide induced chondrocytes injury: an in vitro osteoarthritis model. Molecules.

[CR50] Lingaraj K, Poh CK, Wang W (2010). Vascular endothelial growth factor (VEGF) is expressed during articular cartilage growth and re-expressed in osteoarthritis. Ann Acad Med Singap.

[CR51] Liu X, Corciulo C, Arabagian S, Ulman A, Cronstein BN (2019). Adenosine-functionalized biodegradable PLA-b-PEG nanoparticles ameliorate osteoarthritis in rats. Sci Rep.

[CR52] Loeser RF, Collins JA, Diekman BO (2016). Ageing and the pathogenesis of osteoarthritis. Nat Rev Rheumatol.

[CR53] Malemud CJ (2019). Inhibition of MMPs and ADAM/ADAMTS. Biochem Pharmacol.

[CR54] Mangalampalli B, Dumala N, Grover P (2018). Allium cepa root tip assay in assessment of toxicity of magnesium oxide nanoparticles and microparticles. J Environ Sci (China).

[CR55] Martínez DTI, Vida C, Garrido A, De la Fuente M (2020). Redox parameters as markers of the rate of aging and predictors of life span. J Gerontol A Biol Sci Med Sci.

[CR56] Mathiessen A, Conaghan PG (2017). Synovitis in osteoarthritis: current understanding with therapeutic implications. Arthritis Res Ther.

[CR57] Moeini-Nodeh S, Rahimifard M, Baeeri M, Abdollahi M (2017). Functional improvement in rats’ pancreatic islets using magnesium oxide nanoparticles through antiapoptotic and antioxidant pathways. Biol Trace Elem Res.

[CR58] Morgenstern J, Baumann P, Brunner C, Hubbuch J (2017). Effect of PEG molecular weight and PEGylation degree on the physical stability of PEGylated lysozyme. Int J Pharm.

[CR59] Mukherjee K, Acharya K (2018). Toxicological effect of metal oxide nanoparticles on soil and aquatic habitats. Arch Environ Contam Toxicol.

[CR60] Murugan K, Choonara YE, Kumar P, Bijukumar D, du Toit LC, Pillay V (2015). Parameters and characteristics governing cellular internalization and trans-barrier trafficking of nanostructures. Int J Nanomedicine.

[CR61] Ndrepepa G (2019). Myeloperoxidase-a bridge linking inflammation and oxidative stress with cardiovascular disease. Clin Chim Acta.

[CR62] Noyori K, Koshino T, Takagi T, Okamoto R, Jasin HE (1994). Binding characteristics of antitype II collagen antibody to the surface of diseased human cartilage as a probe for tissue damage. J Rheumatol.

[CR63] Ogunyemi SO, Zhang F, Abdallah Y, Zhang M, Wang Y, Sun G, Qiu W, Li B (2019). Biosynthesis and characterization of magnesium oxide and manganese dioxide nanoparticles using Matricaria chamomilla L extract and its inhibitory effect on Acidovorax oryzae strain RS-2. Artif Cells Nanomed Biotechnol.

[CR64] Pareek V, Bhargava A, Bhanot V, Gupta R, Jain N, Panwar J (2018). Formation and characterization of protein corona around nanoparticles: a review. J Nanosci Nanotechnol.

[CR65] Park JY, Kwon S, Kim SH, Kang YJ, Khang D (2020). Triamcinolone-gold nanoparticles repolarize synoviocytes and macrophages in an inflamed synovium. ACS Appl Mater Interfaces.

[CR66] Pascarelli NA, Moretti E, Terzuoli G, Lamboglia A, Renieri T, Fioravanti A, Collodel G (2013). Effects of gold and silver nanoparticles in cultured human osteoarthritic chondrocytes. J Appl Toxicol.

[CR67] Rhoads JP, Major AS (2018). How oxidized low-density lipoprotein activates inflammatory responses. Crit Rev Immunol.

[CR68] Ribera J, Rodríguez-Vita J, Cordoba B, Portolés I, Casals G, Casals E, Jiménez W, Puntes V, Morales-Ruiz M (2019). Functionalized cerium oxide nanoparticles mitigate the oxidative stress and pro-inflammatory activity associated to the portal vein endothelium of cirrhotic rats. PLoS One.

[CR69] Ridolfi A, Caselli L, Montis C, Mangiapia G, Berti D, Brucale M, Valle F (2020). Gold nanoparticles interacting with synthetic lipid rafts: an AFM investigation. J Microsc.

[CR70] Sadeghi L, Babadi VY, Tanwir F (2018). Manganese dioxide nanoparticle induces Parkinson like neurobehavioral abnormalities in rats. Bratisl Lek Listy.

[CR71] Sansanaphongpricha K, Sonthithai P, Kaewkong P, Thavornyutikarn B, Bamrungsap S, Kosorn W, Thinbanmai T, Saengkrit N (2020). Hyaluronic acid-coated gold nanorods enhancing BMP-2 peptide delivery for chondrogenesis. Nanotechnology.

[CR72] Sarkar A, Carvalho E, D’Souza AA, Banerjee R (2019). Liposome-encapsulated fish oil protein-tagged gold nanoparticles for intra-articular therapy in osteoarthritis. Nanomedicine (Lond).

[CR73] Scanzello CR, Goldring SR (2012). The role of synovitis in osteoarthritis pathogenesis. Bone.

[CR74] Shmagel A, Onizuka N, Langsetmo L, Vo T, Foley R, Ensrud K, Valen P (2018). Low magnesium intake is associated with increased knee pain in subjects with radiographic knee osteoarthritis: data from the Osteoarthritis Initiative. Osteoarthritis Cartilage.

[CR75] Singh N, Savanur MA, Srivastava S, D’Silva P, Mugesh G (2019). A manganese oxide nanozyme prevents the oxidative damage of biomolecules without affecting the endogenous antioxidant system. Nanoscale.

[CR76] Singh P, Kim YJ, Zhang D, Yang DC (2016). Biological synthesis of nanoparticles from plants and microorganisms. Trends Biotechnol.

[CR77] Singh P, Pandit S, Mokkapati V, Garg A, Ravikumar V, Mijakovic I (2018). Gold nanoparticles in diagnostics and therapeutics for human cancer. Int J Mol Sci.

[CR78] Sun H, Liu Y, Bai X, Zhou X, Zhou H, Liu S, Yan B (2018). Induction of oxidative stress and sensitization of cancer cells to paclitaxel by gold nanoparticles with different charge densities and hydrophobicities. J Mater Chem B.

[CR79] Suryavanshi A, Khanna K, Sindhu KR, Bellare J, Srivastava R (2017). Magnesium oxide nanoparticle-loaded polycaprolactone composite electrospun fiber scaffolds for bone-soft tissue engineering applications: in-vitro and in-vivo evaluation. Biomed Mater.

[CR80] Tootoonchi MH, Hashempour M, Blackwelder PL, Fraker CA (2017). Manganese oxide particles as cytoprotective, oxygen generating agents. Acta Biomater.

[CR81] Torzilli PA, Arduino JM, Gregory JD, Bansal M (1997). Effect of proteoglycan removal on solute mobility in articular cartilage. J Biomech.

[CR82] Tuschl K, Mills PB, Clayton PT (2013). Manganese and the brain. Int Rev Neurobiol.

[CR83] Vazquez KJ, Andreae JT, Henak CR (2019). Cartilage-on-cartilage cyclic loading induces mechanical and structural damage. J Mech Behav Biomed Mater.

[CR84] Veronese N, La Tegola L, Caruso MG, Maggi S, Guglielmi G (2019). The association between dietary magnesium intake and magnetic resonance parameters for knee osteoarthritis. Nutrients.

[CR85] Wang D, Lin Z, Wang T, Yao Z, Qin M, Zheng S, Lu W (2016). Where does the toxicity of metal oxide nanoparticles come from: the nanoparticles, the ions, or a combination of both?. J Hazard Mater.

[CR86] Wang Z, Yang J, Yang Y, Pu X, Zhao J, Zhang N (2020). Targeted and combined TPCA-1-gold nanocage therapy for in vivo treatment of inflammatory arthritis. AAPS PharmSciTech.

[CR87] Watanabe K, Shibuya S, Ozawa Y, Nojiri H, Izuo N, Yokote K, Shimizu T (2014). Superoxide dismutase 1 loss disturbs intracellular redox signaling, resulting in global age-related pathological changes. Biomed Res Int.

[CR88] Wathiong B, Deville S, Jacobs A, Smisdom N, Gervois P, Lambrichts I, Ameloot M, Hooyberghs J, Nelissen I (2019). Role of nanoparticle size and sialic acids in the distinct time-evolution profiles of nanoparticle uptake in hematopoietic progenitor cells and monocytes. J Nanobiotechnology.

[CR89] Wu M, Guo H, Liu L, Liu Y, Xie L (2019). Size-dependent cellular uptake and localization profiles of silver nanoparticles. Int J Nanomedicine.

[CR90] Yao H, Xu JK, Zheng NY, Wang JL, Mok SW, Lee YW, Shi L, Wang JY, Yue J, Yung SH, Hu PJ, Ruan YC, Zhang YF, Ho KW, Qin L (2019). Intra-articular injection of magnesium chloride attenuates osteoarthritis progression in rats. Osteoarthritis Cartilage.

[CR91] Yue J, Jin S, Gu S, Sun R, Liang Q (2019). High concentration magnesium inhibits extracellular matrix calcification and protects articular cartilage via Erk/autophagy pathway. J Cell Physiol.

[CR92] Zelzer E, Mamluk R, Ferrara N, Johnson RS, Schipani E, Olsen BR (2004). VEGFA is necessary for chondrocyte survival during bone development. Development.

[CR93] Zeng C, Wei J, Li H, Yang T, Zhang FJ, Pan D, Xiao YB, Yang TB, Lei GH (2015). Relationship between serum magnesium concentration and radiographic knee osteoarthritis. J Rheumatol.

[CR94] Zhang D, Liu D, Zhang J, Fong C, Yang M (2014). Gold nanoparticles stimulate differentiation and mineralization of primary osteoblasts through the ERK/MAPK signaling pathway. Mater Sci Eng C Mater Biol Appl.

[CR95] Zhang L, Su H, Wang H, Li Q, Li X, Zhou C, Xu J, Chai Y, Liang X, Xiong L, Zhang C (2019). Tumor chemo-radiotherapy with rod-shaped and spherical gold nano probes: shape and active targeting both matter. Theranostics.

[CR96] Zheng Q, Fang Y, Zeng L, Li X, Chen H, Song H, Huang J, Shi S (2019). Cytocompatible cerium oxide-mediated antioxidative stress in inhibiting ocular inflammation-associated corneal neovascularization. J Mater Chem B.

[CR97] Zhou HF, Chan HW, Wickline SA, Lanza GM, Pham CT (2009). Alphavbeta3-targeted nanotherapy suppresses inflammatory arthritis in mice. FASEB J.

[CR98] Zhou J, Liu C, Sun Y, Francis M, Ryu MS, Grider A, Ye K (2021). Genetically predicted circulating levels of copper and zinc are associated with osteoarthritis but not with rheumatoid arthritis. Osteoarthritis Cartilage.

